# Targeting the Epithelial Alarmin Pathway with Tezepelumab in Highly Comorbid, Biologic-Experienced Severe Asthma: 52-Week Real-World Outcomes

**DOI:** 10.3390/jcm15082849

**Published:** 2026-04-09

**Authors:** Ruperto González-Pérez, Irene De Lorenzo-García, Hemily Izaguirre-Flores, Héctor González-Expósito, Sara García Gil, Paloma Poza-Guedes

**Affiliations:** 1 Allergy Department, Hospital Universitario de Canarias, 38320 Santa Cruz de Tenerife, Spain; pozagdes@hotmail.com; 2 Severe Asthma Unit, Hospital Universitario de Canarias, 38320 Santa Cruz de Tenerife, Spain; hemilykaterine@hotmail.com (H.I.-F.); hectorbrezo@gmail.com (H.G.-E.); 3 Instituto de Investigación Sanitaria de Canarias (IISC), 38320 Santa Cruz de Tenerife, Spain; 4 Pneumology Department, Hospital Universitario Nuestra Señora de La Candelaria, 38010 Santa Cruz de Tenerife, Spain; irenedelorenzo8@gmail.com; 5 Severe Asthma Unit, Hospital Universitario Nuestra Señora de La Candelaria, 38010 Santa Cruz de Tenerife, Spain; 6 Pneumology Department, Hospital Universitario de Canarias, 38320 Santa Cruz de Tenerife, Spain; 7 Hospital Pharmacy Department, Hospital Universitario de Canarias, 38320 Santa Cruz de Tenerife, Spain; saragarcgil@gmail.com

**Keywords:** severe uncontrolled asthma, asthma phenotypes, TSLP, biologics, tezepelumab, precision medicine

## Abstract

**Background/Objectives:** Severe asthma in routine practice often involves long-standing disease, multimorbidity, and prior biologic failure—settings underrepresented in pivotal tezepelumab trials. This study evaluated 52-week real-world effectiveness and safety of tezepelumab in a highly comorbid, predominantly T2-high, biologic-experienced severe asthma cohort from the Canary Islands. **Methods:** TEZNERIFE is a multicenter, retrospective study including consecutive adolescents and adults with GINA Step 5 severe uncontrolled asthma treated with tezepelumab 210 mg every 4 weeks for 12 months. Clinical outcomes, lung function, type 2 biomarkers, upper airway symptoms, and Biologics Asthma Response Score (BARS) were assessed at baseline, 26 weeks, and 52 weeks. **Results:** Fifty-six patients (mean age 53.5 years, 71% female, mean asthma duration 30 years, 84% T2-high; 71% with ≥1 prior biologic) were analyzed. ACT improved from 11.5 ± 3.7 to 15.9 ± 4.7 at 26 weeks and 17.5 ± 4.7 at 52 weeks (both *p* < 0.0001), while annualized exacerbations declined from 2.79 ± 2.0 to 0.50 ± 0.72 and 0.51 ± 0.89 (both *p* < 0.0001). Maintenance oral corticosteroid dose fell from 10.2 ± 8.3 to 6.9 ± 2.4 mg/day at 52 weeks (*p* = 0.014). FEV1% predicted increased from 69.3 ± 19.2% to 75.3 ± 17.7% and 76.2 ± 20.6% (*p* = 0.004 and *p* = 0.001), and blood eosinophils decreased from 234 ± 231 to 146 ± 120 and 147 ± 110 cells/µL (*p* = 0.001 and *p* = 0.013). At one year, 18.9% and 67.9% were classified as good and intermediate responders by BARS; 13.2% were insufficient responders. Two patients discontinued due to non-serious adverse events, while no treatment-related serious events occurred. **Conclusions:** In this difficult-to-treat, multimorbid, biologic-experienced population, tezepelumab achieved sustained improvements in asthma control, exacerbations, lung function, eosinophilic inflammation, and corticosteroid exposure over 52 weeks, supporting upstream alarmin inhibition as a versatile strategy in complex severe asthma.

## 1. Introduction

Severe asthma (SA) is a heterogeneous and potentially life-threatening condition in which symptoms persist despite optimized, guideline-directed therapy, including high-dose inhaled corticosteroids combined with additional controller medications such as long-acting bronchodilators. Although it affects a minority of individuals with asthma, SA accounts for a disproportionate burden of exacerbations, healthcare utilization, and impaired quality of life [1,2]. Patients frequently experience persistent dyspnea, nocturnal symptoms, and recurrent exacerbations requiring systemic corticosteroids or hospitalization. Pathophysiologically, SA is characterized by chronic airway inflammation, airway hyperresponsiveness, structural remodeling, and mucus hypersecretion, which may ultimately lead to fixed airflow limitation in a subset of patients [3]. Advances in asthma biology have demonstrated that severe asthma comprises multiple inflammatory phenotypes and endotypes rather than a single disease entity [4,5]. This paradigm shift has fostered precision medicine approaches and the development of biologic therapies targeting key type 2 (T2) mediators, including immunoglobulin E (IgE), interleukin (IL)-5, and the IL-4/IL-13 axis. [6]. These treatments have improved outcomes in selected populations; however, a substantial proportion of patients remain uncontrolled, partially responsive, or ineligible for currently available biologics that primarily target downstream T2 pathways [7,8,9,10].

Thymic stromal lymphopoietin (TSLP) has emerged as a pivotal epithelial-derived “alarmin” that links environmental triggers to both T2 and non-T2 airway inflammation. Released by airway epithelial and other structural cells in response to allergens, viral infections, pollutants, and mechanical injury, TSLP initiates and amplifies immune responses through activation of dendritic cells, type 2 innate lymphoid cells, mast cells, and other effector populations [11,12]. This cascade promotes T helper 2 (Th2) differentiation and the production of IL-4, IL-5, and IL-13. Elevated airway TSLP levels correlate with disease severity, persistent inflammation, and impaired lung function despite corticosteroid treatment. Importantly, accumulating evidence indicates that TSLP also contributes to non-allergic and T2-low inflammatory pathways, including Th1- and Th17-mediated responses and airway remodeling, positioning it as an upstream regulator across diverse SA endotypes [13,14].

Tezepelumab, a fully human monoclonal antibody targeting TSLP, inhibits its interaction with the TSLP receptor complex and thereby attenuates downstream inflammatory signaling. In pivotal randomized clinical trials involving patients with severe, uncontrolled asthma, tezepelumab significantly reduced exacerbation rates, improved lung function, and decreased biomarkers of airway inflammation—including fractional exhaled nitric oxide, blood eosinophils, and IgE—across a broad spectrum of baseline T2 biomarker levels [15]. These findings support the concept of TSLP inhibition as a broadly effective therapeutic strategy and expand the biologic armamentarium for severe asthma management [16,17,18,19]. Despite this robust clinical trial evidence, important uncertainties remain. Randomized controlled trials enroll selected populations under standardized conditions that may not fully reflect routine clinical practice. In real-world settings, patients often present with overlapping inflammatory endotypes, comorbidities, variable adherence, and distinct environmental exposures that may influence treatment response [20,21,22].

The Canary Islands represent such a setting, characterized by subtropical climatic conditions and the highest reported national prevalence of asthma and prescription rates of biologic therapies for SA in Spain [23]. Previous regional studies have described overlapping inflammatory subtypes in moderate and SA, reflecting a complex and heterogeneous inflammatory landscape [24,25,26]. However, data evaluating the real-world effectiveness of upstream TSLP inhibition in this population are currently lacking. Against this background, the present study aimed to assess the effectiveness and safety of monthly subcutaneous tezepelumab 210 mg at 26 and 52 weeks in patients with uncontrolled SA across different inflammatory endotypes in daily clinical practice.

## 2. Materials and Methods

### 2.1. Study Design and Setting

This multicenter, retrospective, observational study (TEZNERIFE cohort) assessed the real-world effectiveness of upstream TSLP inhibition with tezepelumab in patients with uncontrolled SA. Participants received tezepelumab 210 mg administered subcutaneously every four weeks (q4w) for 12 consecutive months. Follow-up was conducted at the Severe Asthma Unit and Outpatient Allergy and Pneumology Clinic of Hospital Universitario de Canarias (Tenerife, Spain) and at the Severe Asthma Unit and Outpatient Pneumology Clinic of Hospital Universitario La Candelaria (Tenerife, Spain).

### 2.2. Participants

Eligible patients were adolescents and adults (>12 years) with clinician-confirmed severe refractory asthma according to the 2020 Global Initiative for Asthma (GINA) guidelines. Initiation of tezepelumab required uncontrolled disease, with no limitations on baseline biomarker levels, smoking status, or comorbidities, reflecting routine daily clinical practice. All patients underwent regular clinical follow-up every 3–6 months after biologic initiation. Pregnant or breastfeeding women were excluded. The study protocol was approved by the local Ethics Committee of CEIm Hospital Universitario de Canarias, Tenerife, Spain on 30 October 2025 (Acta 20/2025 Ordinaria VIRTUAL), and written informed consent was obtained from all participants or their legal guardians.

### 2.3. Data Collection and Clinical Outcomes

Data were retrospectively extracted from electronic medical records between January 2024 and January 2026. Collected variables included age, sex, age at asthma onset, atopy status, asthma-related comorbidities, exacerbation frequency, emergency department visits, hospitalizations, maintenance and burst oral corticosteroid (OCS) use, Asthma Control Test (ACT) score, spirometric parameters, fractional exhaled nitric oxide (FeNO), blood eosinophils, and serum IgE levels.

Clinically significant exacerbations were defined as worsening asthma requiring systemic corticosteroids for at least three days (or doubling the maintenance dose in patients on chronic OCS therapy) or leading to emergency department attendance or hospitalization [27]. Exacerbation rates during the 12 months before and after tezepelumab initiation were compared. Corticosteroid dependence was defined as daily OCS use for at least six consecutive months, and pulmonary function testing and FeNO measurements were performed according to global standards, and again compared at baseline, 6 months, and 12 months [28,29]. Both absolute and percent predicted values were evaluated, and only patients with paired measurements at each respective time point were included in longitudinal analyses.

### 2.4. Classification of Inflammatory Endotypes

Inflammatory endotype was determined at biologic initiation by the treating physician through an integrated assessment of clinical characteristics and available biomarkers, consistent with current recommendations [30,31]. In short, T2-high asthma was defined by the presence of at least one of the following: blood eosinophils ≥ 150 cells/μL at baseline or ≥300 cells/μL within the previous 12 months, FeNO ≥ 25 ppb, documented allergic sensitization with elevated total or specific IgE, or atopic comorbidities such as allergic rhinitis, nasal polyposis, or eczema. T2-low asthma was assigned when these features were consistently absent, defined by blood eosinophils < 150 cells/μL, FeNO < 25 ppb, and no evidence of allergic sensitization or atopic disease.

### 2.5. Laboratory and Serological Assessments

Peripheral blood samples were obtained from all participants, coded, stored at −40 °C, and analyzed using standardized laboratory procedures. Blood eosinophil counts were measured using automated hematological analysis. Total and specific IgE concentrations were determined using the Immulite 2000 Immunoassay System (Siemens Healthcare Diagnostics, La Jolla, NY, USA). Sensitization to *Dermatophagoides pteronyssinus* and *Dermatophagoides farinae* was assessed. Total IgE was expressed in IU/mL and specific IgE in kUA/L, with values ≥ 0.35 kUA/L considered positive. Biomarker levels at 26 and 52 weeks were compared with baseline values.

### 2.6. Skin Prick Test

The skin prick test (SPT) was performed according to European Guidelines [32], enclosing a diagnostic panel (Inmunotek, Madrid, Spain) with standardized (*Dermatophagoides pteronyssinus* (*D. pteronyssinus*), and *Dermatophagoides farinae* (*D. farinae*) mite extracts. Histamine (10 mg/mL) and saline were used as positive and negative controls as usual. Following everyday practice, antihistamines were withdrawn a week prior to the SPT, and wheal diameters were immediately measured after 20 min and those greater than 3 mm were regarded as positive.

### 2.7. Definition of Treatment Response

Treatment response was evaluated at 6 and 12 months using the Biologics Asthma Response Score (BARS). The composite score incorporates reduction in exacerbation rate, reduction in daily OCS dose, and improvement in ACT score. Each criterion is scored as 2 (good), 1 (intermediate), or 0 (insufficient response), and the final score is calculated by dividing the total points by the number of applicable criteria. Thresholds for response classification were ≥1.5 for good, 0.5–1.33 for intermediate, and <0.5 for insufficient response [33]. An extended version additionally incorporated lung function improvement. FEV_1_ normalization was defined as an increase of at least 100 mL and attainment of ≥80% predicted when baseline FEV_1_ was <80% predicted. Criteria not applicable at baseline were excluded from the denominator, as elsewhere described [34].

### 2.8. Statistical Analysis

Continuous variables are presented as mean ± standard deviation or median (interquartile range), depending on distribution. Categorical variables are expressed as frequencies and percentages. Normality was assessed using the Kolmogorov–Smirnov test. Longitudinal changes in clinical and functional outcomes were analyzed using paired Student’s *t*-tests or Wilcoxon signed-rank tests, as appropriate, to evaluate within-patient changes over time in this retrospective observational cohort. Given the exploratory real-world nature of the study, the relatively small sample size, and incomplete paired data for some variables, multivariable modeling was not performed. Similarly, no formal correction for multiple comparisons was applied; therefore, all *p*-values should be interpreted as hypothesis-generating rather than confirmatory. Effect sizes were calculated to estimate the magnitude of change. A two-sided *p*-value < 0.05 was considered statistically significant. All statistical analyses were conducted using GraphPad Prism version 10.0.0 (GraphPad Software, La Jolla, CA, USA).

## 3. Results

### 3.1. Demographic and Clinical Characteristics of Subjects at Baseline

**Figure 1:**
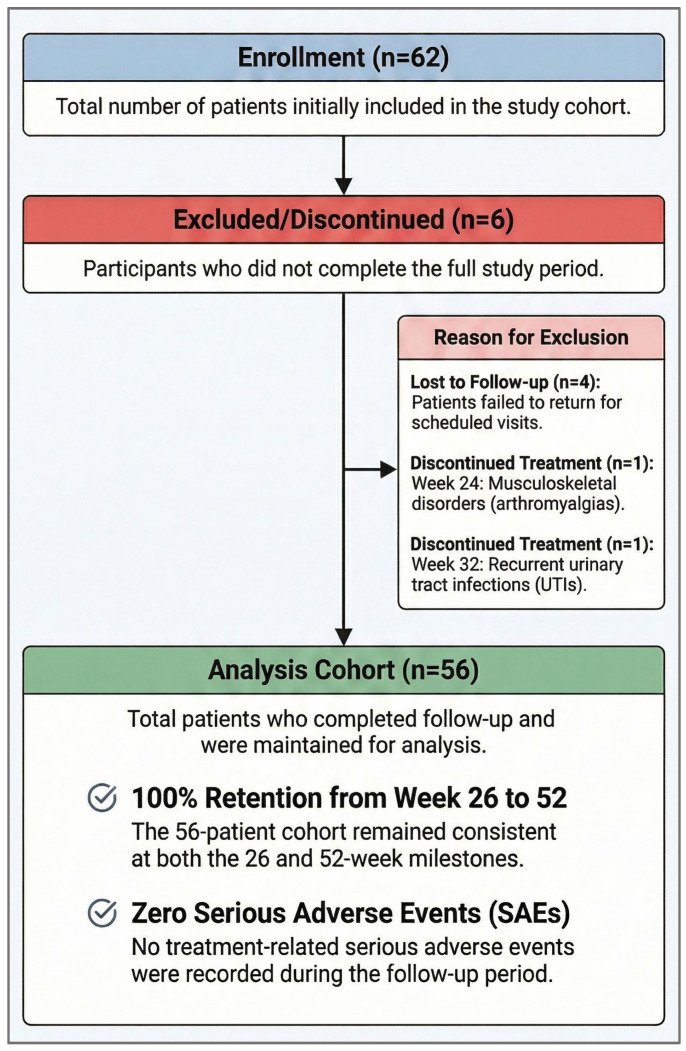
CONSORT Flow diagram of the TEZNERIFE cohort. This diagram illustrates the patient selection process, beginning with 62 initially screened subjects with severe uncontrolled asthma. It details the reasons for exclusion, including four patients lost to follow-up and two patients who discontinued treatment due to specific non-serious adverse events (arthromyalgias and recurrent urinary tract infections), resulting in a final cohort of 56 patients for the 52-week longitudinal effectiveness analysis.

A total of 62 patients were initially included. During follow-up, 4 were lost to follow-up and 2 discontinued treatment (at weeks 24 and 32, respectively), resulting in 56 patients for analysis. This cohort was maintained at both 26 and 52 weeks. Six subjects were subsequently excluded or discontinued: four were lost to follow-up, and two ceased tezepelumab therapy due to adverse events (AEs). Specifically, one patient withdrew at week 24 due to musculoskeletal and connective tissue disorders—arthromyalgias—while the second stopped at week 32 due to recurrent urinary tract infections (Figure 1). No serious AEs related to the treatment were recorded during the study follow-up period.

Therefore, a total of 56 patients with severe uncontrolled asthma receiving biologic therapy were finally included in the study (Table 1). The mean age was 53.47 years (SD 13.3), with 3 patients (5.35%) aged <20 years and 53 (94.64%) aged ≥20 years. The majority were female (71.42%), while 28.58% were male. The mean body mass index (BMI) was 30.1 kg/m^2^ (SD 6.53), indicating that, on average, the population was in the obese range. The mean duration of asthma was 29.98 years (SD 15.44), and 26 patients (46.42%) reported childhood-onset asthma. A family history of atopy was present in 41 patients (80.32%). Most patients exhibited a T2-high inflammatory phenotype (83.82%), whereas 16.08% were classified as T2-low. All patients (100%) were treated at Global Initiative for Asthma (GINA) Step 5, and 6 (10.74%) were receiving maintenance daily oral corticosteroids at baseline.

Regarding prior biologic exposure before initiation of tezepelumab, 16 patients (28.57%) were biologic-naïve. Eighteen (32.14%) had received one prior biologic, 14 (25.0%) had received two, 6 (10.71%) had received three, and 2 patients (3.56%) had received four or more prior biologics (one patient each with four and five previous agents). In terms of smoking status, 37 patients (66.07%) were never smokers, 16 (28.57%) were former smokers, and 3 (5.35%) were current smokers. Bronchiectasis confirmed by chest CT was present in 4 patients (7.1%). Sensitization to house dust mite (HDM) was documented in 25 patients (44.64%). Comorbidities were common. Allergic rhinitis was reported in 27 patients (48.21%), atopic dermatitis in 4 (7.14%), chronic rhinosinusitis with nasal polyposis (CRSwNP) in 5 (8.93%), chronic rhinosinusitis without polyposis (CRSsNP) in 2 (3.57%), and nonsteroidal anti-inflammatory drug-exacerbated respiratory disease (NERD) in 2 (3.57%). Gastroesophageal reflux disease (GERD) was present in 15 patients (26.78%), depression in 9 (16.07%), and obstructive sleep apnea in 13 (23.21%). Overall, the cohort was characterized by long-standing, predominantly T2-high, severe asthma with a high burden of atopy and comorbid conditions.

### 3.2. Asthma Control and Exacerbations

Treatment with subcutaneous tezepelumab 210 mg every 4 weeks was associated with a rapid and sustained improvement in overall asthma control (Table 2).

**Figure 2:**
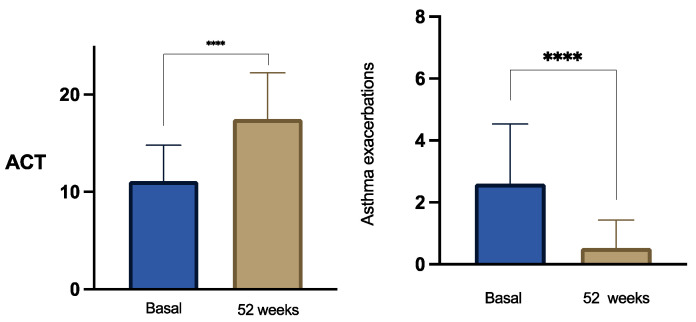
Evolution of asthma control measured by the Asthma Control Test (ACT) in subjects with severe refractory asthma before and after 52-week treatment with tezepelumab 210 mg every 4 weeks. Asterisks indicate statistical significance (**** *p* < 0.001).

Mean ACT scores increased from 11.52 ± 3.67 at baseline (Figure 2), consistent with poorly controlled disease, to 15.89 ± 4.73 at 26 weeks and 17.48 ± 4.73 at 52 weeks (both *p* < 0.0001 vs. baseline). The progressive rise in ACT values over time reflects a clinically meaningful shift toward better symptom control during follow-up. In parallel, exacerbation burden was markedly reduced. The annualized number of asthma exacerbations declined from 2.79 ± 2.0 at baseline to 0.50 ± 0.72 at 26 weeks and 0.51 ± 0.89 at 52 weeks (both *p* < 0.0001). This substantial and sustained reduction underscores the treatment’s effectiveness in preventing acute disease worsening over one year of observation.

### 3.3. Oral Corticosteroid Exposure

Baseline maintenance oral corticosteroid (OCS) use averaged 10.2 ± 8.3 mg/day. A numerical decrease was observed after 26 weeks (8.41 ± 6.2 mg/day; *p* = 0.1831), followed by a statistically significant reduction at 52 weeks to 6.9 ± 2.4 mg/day (*p* = 0.0140 vs. baseline). These findings indicate a delayed but meaningful corticosteroid-sparing effect emerging with longer treatment duration.

### 3.4. Lung Function

Pulmonary function parameters improved during tezepelumab therapy. Mean FVC increased from 2940 ± 992 mL at baseline to 3289 ± 1234 mL at 26 weeks (*p* = 0.0161) and remained elevated at 3214 ± 1148 mL at 52 weeks (*p* = 0.0125).

**Figure 3:**
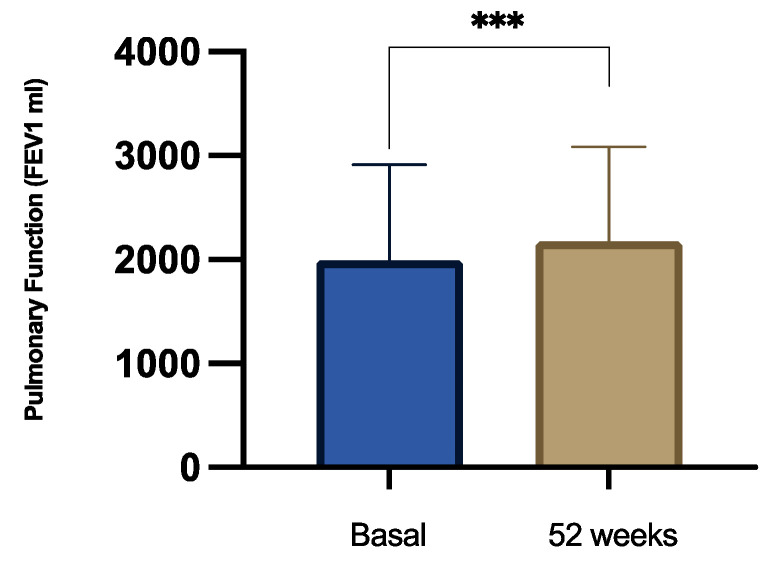
Evolution of pulmonary function (FEV1 in mL) before and after 52-week treatment with tezepelumab 210 mg every 4 weeks. Asterisks indicate statistical significance (*** *p* = 0.0384).

FEV_1_ rose from 2061 ± 895 mL at baseline to 2206 ± 923 mL at 26 weeks (*p* = 0.0572) and further to 2316 ± 958 mL at 52 weeks (Figure 3), reaching statistical significance at one year (*p* = 0.0384). Consistently, FEV_1_% predicted improved from 69.31 ± 19.2% to 75.32 ± 17.68% at 26 weeks (*p* = 0.0043) and 76.24 ± 20.63% at 52 weeks (*p* = 0.0012). Together, these data demonstrate early improvements in ventilatory capacity with sustained gains over 12 months.

### 3.5. Type 2 Inflammatory Biomarkers

Markers of type 2 inflammation showed differential patterns of response. Blood eosinophil counts declined significantly from 234 ± 231 cells/μL at baseline to 146 ± 120 cells/μL at 26 weeks (*p* = 0.0012) and 147 ± 110 cells/μL at 52 weeks (*p* = 0.0125), indicating durable suppression throughout follow-up.

FeNO decreased numerically from 29.17 ± 19.9 ppb to 23.93 ± 15.42 ppb at 26 weeks (*p* = 0.0889) and was 26.04 ± 16.87 ppb at 52 weeks (*p* = 0.1960), without statistical significance. Total IgE levels also declined from 354 ± 549 IU/mL at baseline to 240 ± 382 IU/mL at 26 weeks (*p* = 0.1324) and 234 ± 409 IU/mL at 52 weeks (*p* = 0.2356). Similarly, allergen specific IgE to *D. pteronyssinus* and *D. farinae* showed numerical reductions at 12 months (21.17 ± 33.0 to 17.15 ± 4.32 kUA/L, *p* = 0.1833; and 17.1 ± 30.21 to 13.58 ± 4.7 kUA/L, *p* = 0.1499, respectively), without reaching statistical significance.

### 3.6. Upper Airway Outcomes and Composite Response

Among patients with concomitant chronic rhinosinusitis with nasal polyps (n = 6), SNOT-22 scores decreased from 59.94 ± 19.36 at baseline to 55.67 ± 23.69 at 26 weeks (*p* = 0.5171) and to 46.76 ± 26.8 at 52 weeks (*p* = 0.373). Although statistical significance was not achieved, the reduction at one year exceeded the minimal clinically important difference (>8.9 points), suggesting potential symptomatic benefit in upper airway disease.

The combined Biologics Asthma Response Score (BARS) further characterized treatment response. Mean scores were 0.95 ± 0.53 at 26 weeks and 1.06 ± 0.55 at 52 weeks (*p* = 0.0501). At 26 weeks, 19.64% of patients were classified as good responders (≥1.5), 69.64% as intermediate responders (0.5–1.33), and 10.71% as insufficient responders (<0.5). At 52 weeks, corresponding proportions were 18.86%, 67.92%, and 13.2%, respectively, indicating a predominantly intermediate-to-good response profile maintained over time.

## 4. Discussion

This real-world longitudinal study assessed the effectiveness of tezepelumab in a highly complex population with severe uncontrolled asthma characterized by long-standing disease, substantial comorbidity burden, frequent prior biologic exposure, and universal GINA Step 5 treatment. The cohort reflects a difficult-to-treat phenotype typical of tertiary care settings, including obesity, overlapping upper and lower airway disease, psychiatric and sleep-related comorbidities, and considerable cumulative corticosteroid exposure—features often underrepresented in pivotal trials [15,17]. Within this challenging clinical context, tezepelumab was associated with clinically meaningful improvements across multiple domains over 52 weeks, consistent with emerging real-world data in similarly heterogeneous populations [35,36,37].

Asthma control improved markedly and was accompanied by a profound reduction in exacerbation frequency. At baseline, patients exhibited very poor symptom control (mean ACT 11.5) and frequent exacerbations (mean 2.79/year), reflecting substantial disease instability. Over 12 months, ACT scores increased progressively, while exacerbation rates declined by approximately 80%, with stability between 26 and 52 weeks. These findings are comparable to the 60–70% reductions reported in randomized trials and recent real-world studies [38,39,40]. Given that many patients had previously failed one or more biologic therapies, the magnitude and durability of exacerbation reduction are particularly noteworthy and align with evidence demonstrating benefit in biologic-experienced and low-eosinophil populations.

A corticosteroid-sparing effect further supports the clinical relevance of these outcomes. Although reduction in maintenance OCS dose did not reach statistical significance at 26 weeks, a significant decrease was observed at one year, likely reflecting cautious tapering in patients with long-standing steroid dependence and multimorbidity [18,35,41,42]. Even moderate reductions in chronic OCS exposure are clinically meaningful in this population, given the high prevalence of obesity, GERD, obstructive sleep apnea, and depression—conditions closely associated with cumulative corticosteroid toxicity and identified as dominant traits limiting remission [43,44]. The observed reduction therefore represents an important step toward limiting long-term treatment-related harm [45].

Lung function improved early and was sustained throughout follow-up. FVC increased significantly at both 26 and 52 weeks, while FEV_1_ and FEV_1_% predicted showed progressive gains reaching significance at one year. Considering the nearly three-decade mean disease duration and the presence of structural airway comorbidities in some patients, these changes are clinically relevant [46]. In long-standing asthma, airflow limitation may include a partially fixed component related to airway remodeling; thus, sustained functional improvement suggests that upstream TSLP inhibition may attenuate inflammatory mechanisms contributing to persistent airflow obstruction.

Biomarker analyses provide additional mechanistic insight. Blood eosinophil counts declined significantly and remained suppressed over 12 months, indicating sustained modulation of type 2 inflammation. In contrast, FeNO and total IgE showed only numerical, non-significant reductions. This dissociation between clinical improvement and certain biomarker changes likely reflects biological heterogeneity, including both T2-high and T2-low phenotypes as well as prior exposure to IL-5 or IgE-targeted therapies. Notably, clinical response was not dependent on marked reductions in all conventional type 2 biomarkers, supporting the broader upstream mechanism of TSLP inhibition [47]. In the subgroup with CRSwNP, SNOT-22 scores decreased beyond the minimal clinically important difference at 52 weeks. Although statistical significance was not reached—likely due to limited sample size—the magnitude and direction of change are clinically meaningful. The shared epithelial-driven inflammatory pathways underlying asthma and sinonasal disease provide a biological rationale for parallel improvement under TSLP inhibition, warranting confirmation in larger dedicated studies.

The composite BARS analysis showed that most patients achieved an intermediate or good response, with a stable distribution between 26 and 52 weeks. Nearly one-fifth fulfilled criteria for a good response despite extensive prior biologic exposure, while only a small proportion were classified as insufficient responders. This composite index integrates multiple clinically relevant domains, providing a pragmatic and holistic assessment of treatment effectiveness in routine practice. In this context, its use allows for a more comprehensive evaluation than single-parameter endpoints, particularly in heterogeneous real-world populations. However, although BARS has been applied in clinical studies, its validation across diverse real-world settings remains limited [48]. Therefore, while it offers practical clinical value in assessing response to biologic therapy, the results should be interpreted with caution, and further studies are warranted to confirm its robustness and generalizability. Overall, our findings highlight the broad applicability of tezepelumab across heterogeneous phenotypes and suggest a durable rather than transient therapeutic effect [49]. Safety findings were consistent with established clinical experience [50,51]. Two patients discontinued therapy due to non-serious adverse events, and no treatment-related serious adverse events were reported. In a multimorbid population with high healthcare utilization, this tolerability profile supports the feasibility of long-term use in routine practice.

The study’s strengths include its real-world design and the inclusion of patients with multimorbidity, smoking history, structural lung disease, and prior biologic exposure, enhancing external validity. Predefined longitudinal assessments at 26 and 52 weeks allowed for the evaluation of both early and sustained treatment effects. However, the modest sample size, absence of a control group, and limited statistical power restrict the interpretation of subgroup analyses, particularly for upper airway outcomes and comparisons between biologic-experienced versus biologic-naïve patients and T2-high versus T2-low SA s phenotypes. Residual confounding related to concomitant therapies or comorbidity management cannot be excluded. In addition, the absence of a comparator group precludes causal inference and limits the ability to directly attribute observed changes to tezepelumab. Therefore, the findings should again be interpreted as descriptive of real-world effectiveness. Nevertheless, the consistent improvements observed across symptom control, exacerbation rates, lung function, and eosinophilic inflammation support the robustness of the findings and align with results from larger randomized and real-world studies [39].

## 5. Conclusions

In this difficult-to-treat, clinically heterogeneous severe asthma population with substantial prior biologic exposure and high comorbidity burden, tezepelumab was associated with sustained improvements in asthma control, exacerbation frequency, lung function, eosinophilic inflammation, and corticosteroid exposure over 52 weeks. These data extend randomized trial evidence into a complex real-world setting and support upstream epithelial alarmin inhibition as an effective and versatile strategy in SA management.

## Figures and Tables

**Table 1:** Demographic and clinical characteristics of patients prior to commencement of Tezepelumab 210 mg every 4 weeks.

Variable	Severe Uncontrolled Asthma
N (%)	56 (100)
Age (y.o.) mean (SD)	53.47 (±13.3)
<20 y.o. (%)	3 (5.35)
≥20 y.o. (%)	53 (94.64)
Female Sex (%)	40 (71.42)
BMI mean (SD)	30.1 (±6.53)
Duration of Asthma (y) mean (SD)	29.98 (±15.44)
Asthma Onset at Childhood (%)	26 (46.42)
Family History of Atopy (%)	41 (80.32)
Asthma phenotype (T2 High/T2 Low, %)	83.82/16.08
GINA Step 5 Treatment Level (%)	56 (100)
Daily Oral Corticosteroids (%)	6 (10.74)
Former Use of Severe Asthma Biologics before Tezepelumab (%)	40 (71.4)
None (%)	16 (28.57)
One (%)	18 (32.14)
Two (%)	14 (25.0)
Three (%)	6 (10.71)
Four (%)	1 (1.78)
Five (%)	1 (1.78)
Smoking	
Never smoker (%)	37 (66.07)
Former smoker (%)	16 (28.57)
Smoker (%)	3 (5.35)
Bronchiectasis (Chest CT Scanner) (%)	4 (7.1)
SPT+ HDM (%)	25 (44.64)
Allergic Rhinitis (%)	27 (48.21)
Atopic Dermatitis (%)	4 (7.14)
Chronic Rhinosinusitis with Nasal Polyposis (%)	5 (8.93%)
NERD (%)	2 (3.57)
Chronic Rhinosinusitis (%)	2 (3.57)
GERD (%)	15 (26.78)
Depression (%)	9 (16.07)
Obstructive Sleep Apnea Syndrome (%)	13 (23.21)

SD: Standard deviation of the mean. SPT: Skin Prick Test. HDM: House dust mites (*Dermatophagoides* spp.) NERD: NSAID-exacerbated respiratory disease. GERD: Gastroesophageal reflux disease.

**Table 2:** Longitudinal assessment of patients with severe uncontrolled asthma at baseline and after 26 and 52-week treatment with subcutaneous tezepelumab 210 mg every 4 weeks. SNOT-22 evaluation applies only for patients (n = 6) afflicted with comorbid chronic rhinosinusitis with nasal polyps.

Variables	Baseline	After 26-Weeks of Tezepelumab (n = 56)	*p*	After 52-Weeks of Tezepelumab (n = 56)	*p*
ACT	11.52 ± 3.67	15.89 ± 4.73 *	<0.0001	17.48 ± 4.73 *	<0.0001
Number of annual AEs	2.79 ± 2.0	0.5 ± 0.72 *	<0.0001	0.51 ± 0.89 *	<0.0001
Use of OCS (mg/day)	10.2 ± 0.8.3	8.41 ± 6.2	0.1831	6.9 ± 2.4 *	0.0140
FVC (mL)	2940 ± 992	3289 ± 1234 *	0.0161	3214 ± 1148 *	0.0125
FEV1 (mL)	2061 ± 895	2206 ± 923	0.0572	2316 ± 958 *	0.0384
FEV1%	69.31 ± 19.2	75.32 ± 17.68 *	0.0043	76.24 ± 20.63 *	0.0012
FENO (ppb)	29.17 ± 19.9	23.93 ± 15.42	0.0889	26.04 ± 16.87	0.1960
Blood Eosinophils/μL	234 ± 231	146 ± 120 *	0.0012	147 ± 110 *	0.0125
Total IgE (IU/mL)	354 ± 549	240 ± 382	0.1324	234 ± 409	0.2356
sIgE *D. pteronyssinus* (kU_A_/L)	21.17 ± 33.0	N/A	N/A	17.15 ± 4.32	0.1833
sIgE *D. farinae* (kU_A_/L)	17.1 ± 30.21	N/A	N/A	13.58 ± 4.7	0.1499
SNOT-22	59.94 ± 19.36	55.67 ± 23.69	0.5171	46.76 ± 26.8	0.373
Combined BARS	-	0.95 ± 0.53	-	1.06 ± 0.55	0.0501

ACT: Asthma Control Test. AEs: Asthma exacerbations. OCS: Oral corticosteroids. FVC: Forced Ventilatory Capacity. FEV1: Forced expiratory volume in the first second. SNOT-22: Sino-Nasal Outcome Test-22. FENO: Fractional exhaled nitric oxide. sIgE: Specific IgE. N/A: Not Assessed. BARS: Biologics Asthma Response Score. (*) Indicates statistical significance (*p* < 0.05). Mean values and standard deviation are shown.

## Data Availability

The data that support the findings of this study are available from Servicio Canario de Salud but restrictions apply to the availability of these data, which were used under license for the current study, and so are not publicly available. Data are however available from the authors upon reasonable request and with permission of Servicio Canario de Salud.

## Abbreviations

The following abbreviations are used in this manuscript:

SA	Severe Asthma
TSLP	Thymic stromal lymphopoietin
CRSwNP	Chronic Rhinosinusitis with Nasal Polyposis
BARS	Biologics Asthma Response Score
OCS	Oral Corticosteroid
FeNO	Fractional exhaled Nitric Oxide
